# Case Report: Medullary Thyroid Cancer Workup Initiated by Unexpectedly High Procalcitonin Level—Endocrine Training Saves Life in the COVID-19 Unit

**DOI:** 10.3389/fendo.2021.727320

**Published:** 2021-10-11

**Authors:** Livia Sira, Zoltán Balogh, Eszter Vitális, Dávid Kovács, Ferenc Győry, Csaba Molnár, Miklos Bodor, Endre V. Nagy

**Affiliations:** ^1^ Division of Endocrinology, Department of Medicine, Faculty of Medicine, University of Debrecen, Debrecen, Hungary; ^2^ Division of Infectious Diseases, Faculty of Medicine, University of Debrecen, Debrecen, Hungary; ^3^ Department of Surgery, Faculty of Medicine, University of Debrecen, Debrecen, Hungary; ^4^ Department of Pathology, Faculty of Medicine, University of Debrecen, Debrecen, Hungary

**Keywords:** medullary thyroid cancer, endocrine rotation, calcitonin, procalcitonin, severe acute respiratory syndrome coronavirus 2

## Abstract

**Background:**

Severe acute respiratory syndrome coronavirus-2 (SARS-CoV-2) is a novel coronavirus that has caused a worldwide pandemic. The majority of medullary thyroid cancers present as a thyroid nodule. At the time of diagnosis, cervical lymph nodes and distant metastases are frequently detected.

**Case Report:**

Here, we present a case of a 46-year-old man with coronavirus disease (COVID) pneumonia, who had persistently high serum procalcitonin levels despite normal C-reactive protein levels. The attending infectologist happened to be a colleague who spent some time, as part of her internal medicine rotation, in the Endocrine Ward and recalled that medullary thyroid cancer might be the cause. This led to the timely workup and treatment of the medullary cancer.

## Introduction

The severe acute respiratory syndrome coronavirus 2 (SARS-CoV-2) has resulted in an ongoing pandemic that has posed an extraordinary challenge for healthcare systems worldwide. Among other biomarkers, serum procalcitonin (PCT), C-reactive protein (CRP), ferritin, D-dimer, interleukin-6, and lactate dehydrogenase (LDH) are associated with disease severity and mortality. It is widely accepted that the increase in PCT level indicates the beginning of a critical phase of viral infection (1). In Chinese hospitalized coronavirus disease 2019 (COVID-19) patients, elevated PCT (≥0.10 ng/ml) and CRP levels were independent risk factors of mortality with hazard ratios of 52.68 and 5.47, respectively (2). In contrast, neutrophil-to-lymphocyte ratio (≥3.59) was not found to be an independent risk factor for death in this study. PCT, as a calcitonin-related gene product, is a calcitonin precursor expressed by human epithelial cells during severe systemic inflammation, infection, and sepsis. However, PCT is also the pro-hormone of calcitonin and is co-secreted with calcitonin by thyroid parafollicular cells. PCT has recently emerged as a biomarker of medullary thyroid cancer (MTC) (3–5). The measurement of serum calcitonin level reflects the number and activity of normal parafollicular and MTC cells. Serum calcitonin concentrations in patients with thyroid nodules can lead to an early diagnosis of MTC.

MTC accounts for about 5% of all thyroid malignancies (6). Carcinoembryonic antigen (CEA) is a less specific biomarker for MTC than calcitonin and PCT, but can be useful in the estimation of disease extension and prognosis. After surgery, the doubling times of calcitonin and CEA serum levels are predictive of both recurrence-free survival and overall survival (7). The follow-up of patients with undetectable tumor markers includes annual measurements of serum calcitonin and CEA levels (8).

## Case Presentation

A 46-year-old Caucasian man has been hospitalized in the Coronavirus Ward of the University of Debrecen Clinical Center in January 2021. He presented after 5 days of high fever, dry cough, nausea, and muscle pain. Both PCR and antigen tests confirmed SARS-CoV-2 infection. He has had a history of hypertension and type 2 diabetes known for 4 years and has been on a diet and metformin with recent HbA_1c_ level in the target range. Physical examination showed crepitation on the base of the right lung. Vital parameters were as follows: body temperature 36.7°C, blood pressure 134/94 mmHg, pulse rate 75/min, O_2_ saturation 97%–99%. On admission, CRP was 5.2 mg/L (reference range <5.0 mg/L) and procalcitonin (PCT) was 5.4 ng/ml (reference range <0.50 ng/ml). Quantitative and qualitative blood counts were normal. Chest computed tomography revealed irregularly shaped ground-glass opacities (GGOs) affecting the right lower lobe; lung parenchymal involvement was estimated to be 5%.

Antiviral treatment was started: favipiravir (Zhejiang Hisun Pharmaceutical, China) 1.6 g twice daily on day 1, followed by 600 mg twice daily for a total duration of 5 days. There was no need for O_2_ therapy. His diabetes was well-managed with diet and metformin. During his hospital stay, he received low-molecular weight heparin for the prophylaxis of thromboembolism. CRP remained low at 3.6 mg/L the day after admission, and no decline in the PCT level was detected (6.06 ng/ml). The consistently normal CRP with persistently high PCT levels could not be explained by infection. The infectologist (EV) who has seen endocrine patients during rotation in the internal medicine ward was seeking advice from an endocrinologist. Additional tests for MTC were performed; serum calcitonin and CEA levels were found to be 89.0 ng/L (reference range <11.8 ng/L) and 7.5 µg/L (reference range <3.4 µg/L), respectively.

Ultrasound of the neck region revealed normal sized thyroid with regular echostructure. Two small hypoechoic nodules, with largest diameters of 9 and 5 mm, were detected in the left lobe; no suspicious lymph nodes were seen in the neck region. Computed tomography (CT) demonstrated several 5–10-mm lymph node metastases on the left side of the neck, while no remarkable findings were seen in the chest. Fatty liver disease was suspected based on abdominal ultrasound.

On-site frozen section histology confirmed MTC in a 14-mm lymph node removed from the region of the left jugular vein (level III). Total thyroidectomy was performed. Lymph nodes were excised from the central neck region (level VI) with central neck dissection (CND) and from the left lateral neck region (levels IIA, III, IV) with modified radical neck dissection (MRND).

**Figure 1:**
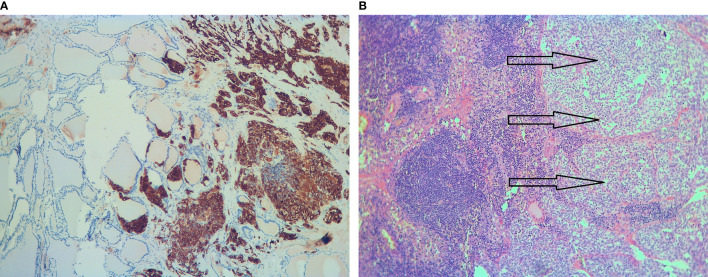
Histology of the surgical specimen. **(A)** Calcitonin-positive medullary thyroid cancer is adjacent to normal thyroid tissue (immunohistochemistry, ×10). **(B)** Solid nests of metastatic medullary thyroid cancer (arrows) in a lymph node (hematoxylin and eosin, ×10).

Final histology revealed three malignant foci (8, 4, and 2 mm in diameter) in the left thyroid lobe. The right lobe was tumor-free. Tumor cells were positive for calcitonin, chromogranin-A, synaptophysin, and neural cell adhesion molecule (CD56) by immunohistochemistry (**Figure 1**). The MIB-1 proliferation marker was positive in 2%. Vascular and lymphatic invasion was detectable, while extrathyroidal invasion was not confirmed. In six of the 16 lymph nodes removed, metastatic tissue was detected (one central and five near the left jugular internal vein). The largest metastasis was 9 mm in diameter with extranodal spread, stage pT1a (m), pN1b according to TNM classification (9).

Follow-up values 4 weeks after surgery were as follows: serum calcitonin 11.3 ng/L (normal <11.8 ng/L), PCT 0.28 µg/L (normal <0.50 µg/L), CEA 1.8 µg/L (normal <3.4 µg/L), total calcium 2.51 mmol/L (normal range 2.10–2.60 mmol/L), phosphate 1.05 mmol/L (normal range 0.8–1.45 mmol/L). On 150 µg levothyroxine supplementation, thyroid-stimulating hormone (TSH) was 0.612 mU/L (normal range 0.30–4.2 mU/L).

The patient has been screened for clinical features of the MEN-2 syndrome, including 24-h urine metanephrines, serum calcium, ionized calcium, and parathormone measurements. His family history for MTC and MEN-2 components was negative. Rearranged during transfection (RET) mutational analysis did not reveal mutations in codons Cys609, Cys611, Cys618, Cys620, and Cys 634.

## Discussion

The extreme burden of the COVID-19 epidemic results in overloaded medical systems and may lead to burn out of the overworked health care personnel. In the case presented here, the high PCT value detected on admission led to the hospitalization of the patient with mild COVID-19 infection and resulted in incidental detection of his MTC. On the day of admission, the attending infectologist happened to be a colleague who spent some time, as part of her internal medicine rotation, in the Endocrine Ward. When looking at the unusual combination of high PCT and near-normal CRP, she recalled that MTC might be the cause. Timely workup and treatment of the underlying MTC were successful (**Table 1**). Recently, a similar case of a 43-year-old man affected by SARS-CoV-2 pneumonia with persistently high PCT levels, and subsequently diagnosed with MTC, has been reported (10).

**Table 1:** Diagnosis and treatment of medullary cancer in our case.

5 January 2021	Confirmed SARS-CoV-2 infection, CRP and PCT measurement
8 January 2021	CEA and calcitonin measurement
11 January 2021	Ultrasound of the neck region
29 January 2021	Computed tomography of neck and chest
10 February 2021	Total thyroidectomy
April 2021	RET mutational analysis

The 8- and 4-mm cancer foci on final histopathology corresponded to the 9- and 5-mm nodules detected by ultrasonography (US), while the 2-mm MTC focus was not detected by US. Fine-needle aspiration (FNA) was not performed, as our institutional protocol calls for thyroidectomy regardless of the FNA result if a serum calcitonin level above 5× upper limit of normal (ULN) in a patient with normal renal function is combined with CEA elevation. Of the imaging performed, the chest CT was part of the COVID workup on admission. The neck CT performed after the neck ultrasound may have been superfluous; although it revealed unexpected new information, it did not change the therapeutic decision. Furthermore, at CT, the largest lymph node was 10 mm, but at on-site histology, it was 14 mm. According to the detailed final histology report, the diameter of the metastasis was 12 mm in the 14-mm lymph node. All these point to certain inaccuracies in size measurement and call for improvement at our institution.

One limitation of this case report is that we cannot provide a cutoff of PCT in detecting MTC in patients with COVID-19. Furthermore, there is still no agreement in the literature on the serum calcitonin cutoff to exclude MTC. In contrast to the 10-min half-life of calcitonin, PCT has a 22-35 hour half-life in serum (10).

There are two ways how the coronavirus epidemic may interfere with thyroid care. On the one hand, COVID-19-related thyroid disorders can manifest as thyrotoxicosis, hypothyroidism, or non-thyroidal illness syndrome (11). On the other hand, elective thyroid surgery is often delayed despite the low risk of SARS-CoV-2 transmission during and after thyroid surgery. A recent systematic review concluded that urgent surgery should be considered for thyroid cancers that exhibit aggressive tumor biology or local invasion (12). We can only speculate on how the natural course of the MTC would have proceeded without the early detection of the disease. As this was a mild COVID-19 case, which usually does not require hospitalization, the persisting high PCT level would have most probably evoked repeated testing by the family doctor after a few weeks. During the epidemic, access to non-COVID medical care is less instantaneous, which may have unfavorable effect on the course of malignant diseases.

## Conclusion

SARS-CoV-2 infection and a disproportionately high level of PCT, relative to CRP, aided in the rapid detection and treatment of MTC. The endocrine training of the attending infectologist led to this unexpected benefit.

## Data Availability Statement

The original contributions presented in the study are included in the article/supplementary material. Further inquiries can be directed to the corresponding author.

## Ethics Statement

Ethical review and approval were not required for the study on human participants in accordance with the local legislation and institutional requirements. The patient/participant provided a written informed consent to participate in this study.

## Author Contributions

LS interpreted the patient data and did the literature review. LS and EN were major contributors in planning treatment and preparation of the manuscript. EV treated the COVID-19 symptoms and recognized the endocrinological disturbances. CM performed the pathological examination of tissue samples. FG and DK operated on the patient. ZB, FG, DK, and MB helped analyze patient data and draft the article. All authors contributed to the article and approved the submitted version.

## Conflict of Interest

The authors declare that the research was conducted in the absence of any commercial or financial relationships that could be construed as a potential conflict of interest.

## Publisher’s Note

All claims expressed in this article are solely those of the authors and do not necessarily represent those of their affiliated organizations, or those of the publisher, the editors and the reviewers. Any product that may be evaluated in this article, or claim that may be made by its manufacturer, is not guaranteed or endorsed by the publisher.
